# When a dying patient is asked to participate in a double-blind, placebo-controlled clinical trial on symptom control: The decision-making process and experiences of relatives

**DOI:** 10.1177/02692163221127557

**Published:** 2022-12-12

**Authors:** Harriëtte J van Esch, Arianne Stoppelenburg, Lia van Zuylen, Carin CD van der Rijt, Agnes van der Heide

**Affiliations:** 1 Department of Medical Oncology, Erasmus MC Cancer Institute, University Medical Center Rotterdam, Rotterdam, The Netherlands; 2 Laurens Cadenza, Rotterdam, The Netherlands; 3 Department of Public Health, Erasmus MC, University Medical Center Rotterdam, Rotterdam, The Netherlands; 4 Department of Medical Oncology, Amsterdam University Medical Centers, Cancer Centrum Amsterdam, Amsterdam, The Netherlands

**Keywords:** End of life care, research design, research subject, family

## Abstract

**Background:**

Placebo-controlled trials can provide evidence to inform end-of-life care, but it is contested whether asking dying patients to participate in such trials is morally justifiable. To investigate the experiences of these patients is even more complex. Therefore, proxy assessments by relatives can be a good alternative.

**Aim:**

To explore the experience of participating in a placebo-controlled trial at the end of life from the perspective of bereaved relatives.

**Design:**

Mixed-method study, including questionnaires and interviews.

**Setting/participants:**

The SILENCE study was a randomized, double-blind, placebo-controlled trial on the efficacy of scopolamine butylbromide to prevent death rattle. The study was performed in six inpatient hospice facilities. Patients were asked to participate at admission in the hospice. Three months after the death of the patient, bereaved relatives were invited to fill in a questionnaire and to participate in an interview. One hundred four questionnaires were completed and 17 relatives were interviewed.

**Results:**

Fourteen percent of the relatives participating in the questionnaire study considered the participation of their loved one in research a bit burdensome and 10% considered it a bit stressful. Seventeen percent thought that it was a bit burdensome for the patient. Eighty-three percent considered participation in this type of research (very) valuable. The in-depth interviews showed that patients and relatives jointly decided about participation in this double-blind placebo-controlled medication trial. Relatives generally respected and felt proud about patients’ decision to participate.

**Conclusion:**

The large majority of bereaved relatives experienced the participation of their dying love one in this RCT as acceptable and valuable.

**What is already known?**
At the end of life, most patients and relatives are willing to participate in research.
There are no studies on the experiences of relatives when their dying loved one is asked to participate in a randomized placebo-controlled clinical trial.
Little is known about the decision-making of patients who are nearing death about participating in research.
**What this paper adds?**
Patients decide about participating in end-of-life care research in close consultation with their relatives.
Participation in a randomized placebo-controlled medication trial at the end of life was not experienced as burdensome by most relatives.
**Implications for practice**
The results of this study show that RCTs at the end of life can be performed without significantly burdening relatives.
Informed consent procedures should allow patients to make well-considered decisions about participation together with their relatives.

## Introduction

There is a need to generate more evidence on symptom treatment in the dying phase.^1^ Randomized controlled trials (RCTs) can contribute to such evidence, but this type of studies are rarely performed among patients who are at the end of life. Many issues, such as patients’ potential vulnerability, obtaining informed consent and gatekeeping by health care professionals, complicate the design, and execution of RCTs among patients who are at the end of life.^2–4^ However, many patients with advanced illness are willing to participate in research.^5,6^ A review conducted in 2018 found that the main reasons for patients to participate in end-of-life research are the desire to contribute to science, the desire to help others and the possibility that they will personally benefit from participation.^5^ Patients’ willingness to participate in research seems to depend on the burden of the activities that are needed when participating in the study. Studies involving completion of short questionnaires, or additional diagnostic investigations or hospital visits, as well as medication studies, seem to be acceptable for most patients.^5,7^

A few studies have examined the experiences of relatives of patients who participate in end-of-life research. In most of these studies, relatives themselves were active research participants.^8,9^ They participate for mostly the same reasons as patients. One study found that relatives experienced a direct benefit of participating in research: it helped them in coping with their loved one’s illness and the nearing end of life.^9^

It is complex to investigate the experience of dying patients, in particular regarding a symptom such as death rattle. Proxy assessments by relatives can be a good alternative.^10^ Involving relatives in all phases of palliative care is essential as palliative care is aimed at improving quality of life of patients and their families.^11^ Therefore, it is important to study whether and how participation in research at the end of life affects the dying phase according to the relatives. It has been shown that relatives have an active role involving medical decision making at the end of life.^12^ However, there are, to our knowledge, no studies on the experiences of relatives of the involvement of their dying loved one in research where they do not also actively participate themselves. Further, little is known about how imminently dying patients decide to participate in research.

Better understanding of the decision making on participation in end-of-life care research may improve informed consent procedures. We performed a study to assess the experiences of relatives of imminently dying patients participating in a randomized placebo-controlled trial.

## Methods (706)

### Study design

We performed a study with a mixed-methods design: a combination of a questionnaire study and an interview study among bereaved relatives of patients who died in a hospice after they had participated in the SILENCE study. The SILENCE study was a randomized double-blind, placebo-controlled trial on the efficacy of prophylactically given scopolaminebutyl for death rattle. The design of this study and its results have been published elsewhere.^13,14^ Combining questionnaire and interviews can provide in-depth understanding of the lived experiences of relatives. The study was approved by the Medical Ethical Research Committee of Erasmus MC, University Medical Center Rotterdam (MEC-2016-429; 29th December 2016).

### Setting and procedures

The SILENCE study was performed in six inpatient hospice settings. In the Netherlands, patients who are admitted to a hospice are expected to have a life expectancy of less than 3 months and to stay there until they die.

On admission, patients were informed about the study by the hospice doctor and received a written information letter. The patient was asked for written informed consent to participate in the trial. Furthermore, the relative who was the first contact person for the hospice was asked if he/she would be willing to receive a questionnaire 3 months after the patient’s death. Refusal or consent by the relative was registered in the patient’s medical record. Three months after the death of the patient, relatives who had consented received the questionnaire with an accompanying letter explaining the purpose of the study. If there was no response after 2 weeks, a reminder was sent.

The questionnaire included a question whether relatives would be willing to explain their answers in a personal interview. Relatives who positively answered to the question “Are you willing to explain your answers from the questionnaire in a personal interview?” were contacted by telephone by the first author (HVE) at least 6 months after the death of the patient and were informed about the purpose and content of the interview. If relatives still wanted to participate, an appointment for an interview was made.

### Measurements

The questionnaire included questions on demographic characteristics of the relative, such as age, gender, health status and relationship to the patient. The questionnaire also included self-developed questions about the experience and meaning of participation of the patient in the RCT and the burden and stress of such participation for the patient, relative, and other family. A four-point Likert scale (very burdensome/stressful, burdensome/stressful, a bit burdensome/stressful, not burdensome/stressful at all) was used to assess these experiences. Finally, relatives were asked if they themselves would participate in end-of-life research (yes/no) (see Supplemental Appendix 1)

The interviews were conducted by the first author (HVE) at the relative’s home. First, verbal consent was asked from the participants. They were informed that all shared information would be treated confidentially and that reports on the study would be anonymized. The interviews were structured using an interview guide (see Supplemental Appendix 2). The following topics were discussed: (1) evaluation of the patients’ dying phase including the participation in this RCT (e.g. how do you look back on the last days of your loved one’s life? How did you experience the information and communication about the dying phase and this RCT); and (2) the patient’s decision to participate in this RCT and the meaning and experience of this decision for the relative. Questions were asked literally and if necessary additional prompt questions could be used. Interviews were transcribed verbatim. Interviews were held with all relatives who agreed to participate.

### Data analysis

All questionnaire data are presented in descriptive statistics.

Interview transcripts were read and analyzed using the template analysis method with a phenomenological interpretive approach to focus on how the phenomenon is experienced at the time that it occurs, rather than what is thought about this experience or the meaning attributed to it later.^15,16^ The first author (HVE) and the second author (AS) each independently coded the first two interviews and identified initial themes (open coding). The themes were arranged in a code tree using the constant comparative method (axial coding). All authors discussed and agreed on this code tree. Subsequently, the first author (HVE) coded all other interviews using this code tree. The findings were ordered according to two main themes (selective coding) (see Supplemental Appendix 3).

## Results

### Study population

**Figure 1.**
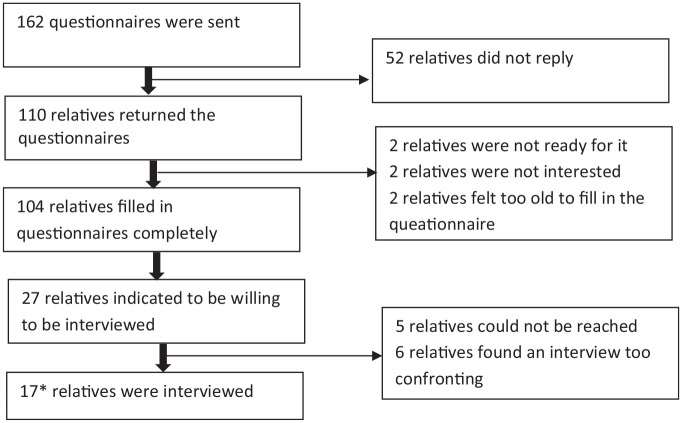
Flowchart inclusion. *Seventeen relatives of 16 patients.

From April 2017 till December 2019, 162 patients participated in the SILENCE study; for all patients, relatives received a questionnaire after the patient died. One hundred ten questionnaires were returned of which 104 (64%) were filled in (Figure 1). Characteristics of these 104 relatives are presented in Table 1. Respondents were more often female than male (59%); they were mostly children and spouses of the patient and their mean age was 57.6 years (SD 12.52).

**Table 1.** Characteristics of the relatives who participated in the questionnaire study (*n* = 104).

	Number (%) *N* = 104
Gender (female)	61 (59)
Age (mean, SD)	57 (12.5)
Relation to patient	
Spouse	20 (19)
Child	59 (57)
Parent	1 (1)
Sibling/cousin/grandchild	23 (22)
Missing	1 (1)

Twenty-seven relatives were willing to participate in an interview. Five could not be reached and six declined at the moment they were invited to make an appointment because they found an interview was emotionally difficult at that time. In the end, 17 relatives of 16 patients were interviewed (Figure 1) in the period from January 2019 till July 2020. In the last three interviews, no new themes were found. Characteristics of the relatives who participated in the interviews are presented in Table 2.

**Table 2.** Relation of relatives who participated in the interview to the patient, patient’s age range, and time between patients’ death and interview.

	*n*
Relation of relative to the patient (*n* = 17)*	
Spouse	4
Son	4
Daughter	6
Other	3
Patient’s age (range) (*n* = 16) 50–59	1
60–69	3
70–79	3
80–89	4
90–99	5
Time between interview and death (months) (=16) 5	1
6	3
7	3
8	3
9	2
10	1
11	1
12	2

* Seventeen relatives of 16 patients were interviewed.

### The experience of relatives with the participation of their loved one in an RCT

The results of the questionnaire are presented in Table 3. The majority of the relatives considered participation of their loved ones in this RCT not burdensome or stressful (*n* = 85 (82%) and *n* = 90 (87%), respectively). A minority of the relatives considered the participation a bit burdensome and/or a bit stressful for themselves (*n* = 6 (14%) and *n* = 4 (10%), respectively), or for other relatives (*n* = 6 (13%) and *n* = 10 (10%), respectively). Twenty (19%) relatives indicated that participation had been (a bit) burdensome for the patient. One relative, however, answered that participation had been very burdensome for the patient. Relatives mostly thought that participation in the RCT had not changed or had improved the quality of dying (*n* = 37 (36%) and *n* = 47 (45%), respectively). Eighty-seven (83%) relatives considered participation in research in general as (very) valuable and 63 (61%) relatives would be willing to participate in research at the end of life themselves.

**Table 3.** Experiences of relatives whose loved ones participated in an RCT on the effect of scopolamine butylbromide on death rattle (*n* = 104).

Meaning and experience	*n* = 104 (%)
How burdensome was participation of your loved one in the study for yourself?	
Not at all burdensome	85 (82)
A bit burdensome	15 (14)
Burdensome	2 (2)
Very burdensome	1 (1)
Missing	1 (1)
How stressful was participation of your loved one in the study for yourself?	
Not at all stressful	90 (87)
A bit stressful	11 (11)
Stressful	1 (1)
Very stressful	2 (2)
How burdensome was participation of your loved one in the study for other relatives (children/siblings/friends)?	
Not at all burdensome	81 (77)
A bit burdensome	13 (13)
Burdensome	2 (2)
Very burdensome	-
Missing	8 (8)
How stressful was participation of your loved one in the study for other relatives (children/siblings/friends)?	
Not at all stressful	83 (79)
A bit stressful	10 (10)
Stressful	1 (1)
Very stressful	-
Missing	10 (10)
How burdensome do you think participation in the study was for your loved one?	
Not at all burdensome	78 (75)
A bit burdensome	18 (17)
Burdensome	2 (2)
Very burdensome	1 (1)
Missing	5 (5)
Has the quality of dying changed as a result of participation in the study?	
Strongly improved	7 (7)
Improved	37 (36)
Unchanged	47 (45)
Deteriorated	2 (2)
Missing	11 (10)
Do you find participation in research generally valuable?	
Not at all valuable	6 (6)
A bit valuable	8 (8)
Valuable	43 (41)
Very valuable	44 (42)
Missing	3 (3)
Would you participate in future research in the last phase of your life? *n* (%)	
No	8 (8)
Yes	63 (61)
Do not know	32 (30)
Missing	1 (1)

In the interviews, the relatives were asked about burden or stress during the study, about information about starting the medication, and about the impact of participating in the study on the quality of dying.

Relatives explained why participation of their loved one in the RCT had not been stressful or burdening.

*“. . .The care was perfect and this research was unnoticed integrated into this care. No, I didn’t notice anything of it (the research - HVE).” relative 14*

Other relatives explained why they experienced some burden or stress for themselves or their loved one:

*“ I asked someone at the time she was rattling so badly ‘does anyone know if this is a placebo or if this is a real drug’, because . . . well I can’t imagine that it was the real drug, because that sound is so intense.” relative 13*

Relatives were not always informed about the start of study medication. This did not seem to be problematic.

*“No, as I said, the study actually went unnoticed by us.” relative 15*

Regarding the effect of participation in the RCT on the quality of dying, one of the relatives stated:

*“. . .No, I don’t believe that, no. . … Afterwards you naturally think it over. . ..But I don’t believe it has done any harm to her, or prolonged the dying, I absolutely don’t believe that. ” relative 9*

One respondent indicated that care in general is improved through research:

*“It is very nice that you have some more guidelines about what is evidence-based, what really works. So I think this research contributes greatly to quality, yes. ” relative 3*

## Making the decision to participate in the RCT

This theme was only addressed in the interview study. Relatives were asked about the process of decision-making to participate in the study, what motivations there were for participating, and how the relatives viewed the decision.

Patients made the decision to participate in the study mostly after consultation with their relatives.

*“My mother told us: “they asked me to participate in a study.” So, she gave us the papers (the information leaflet - HVE): “Look through it, what do you think, should I do it or not?”. . … She wanted to participate herself and actually looked for confirmation from us whether we thought it sensible or possibly unwise.” relative 11*

Some patients decided on their own.

*“She never really talked about it. She did it for others. . .. . . that was how she was:‘if I can do something, I’ll do it.’” relative 10*

Motivation for participation arose from wanting to contribute to research, wanting to do good for others, and from the hope of gaining benefit from participation. Relatives admired and respected the choice of the patients.

*“When the question to participate came, she said very clearly: oh yes! A research study, yes, I will participate in that! It felt very good for me, because - I know - also professionally - how important research is. And my brother who was there too, he was also very proud that she did that.” relative 4*

Some patients made the decision very quickly with their relatives due to earlier experiences with the sound of death rattle.

*“Because of my father we knew what death rattle was like. . . . so we said yes right away when my husband was admitted to the hospice. If we had not known what death rattle was like, we would have thought more about it. ” relative 5*

Some relatives experienced some uncertainty about participation in the research and also noticed some uncertainty about participating in their loved one.

*“. . .. . ..My mother often repeated, that she wouldn’t want to suffer extra as a result. That was very important to her. And, when the decision was taken - or she herself actually took the decision - she also mentioned ‘if it really goes wrong, then they help you anyway, even if you’re in the placebo group’. . .Yes, she was concerned. . …, she actually wanted the confirmation: if I’m in the wrong group so to speak, they won’t let me down, would they?” relative 11*

One relative was unpleasantly surprised when her mother was asked to participate the RCT upon her admission to the hospice.

*“. . .. . .I found it quite intense. . . .The moment a doctor asks if you want to participate in research, at admission to the hospice, that’s pretty confronting. So, she said: ‘Well, may I think about that for a moment, because I’m receiving so much information.’. . .. . . You enter the hospice . . .. and pretty soon you’re asked that question.” relative 16*

## Discussion

This study showed that the participation of patients in an RCT to assess the effect of scopolamine butylbromide on death rattle was not considered burdensome by the majority of relatives. They considered the study valuable. We also found that according to relatives, participation in the RCT had no impact on the quality of dying of patients. These results suggest that RCTs at the end of life are in principle feasible.

A minority of the relatives had experienced some burden as a consequence of their loved ones’ participation in this study. One patient had experienced some burden in the time between the informed consent and the actual start the study medication according to the relative. This may have been caused by the “advance consent” procedure that was used in the SILENCE study: patients were asked to participate upon admission and the study started when the dying phase was recognized. As a result, a period of time passed allowing patients to reflect with their relatives on the study and their consent. This highlights the need for the repeated provision of personalized information, especially in research using an “advance consent” procedure, but probably in all types of research.^17^

Few patients in this study made the decision to participate in this study on their own, most consulted their relatives. Other studies have also found that the opinions of relatives are highly valued by patients, especially when decisions must be made related to treatment at the end of life.^18^ Although involving relatives in the decision making can be reassuring for patients, they may also play a role in unwarranted “gate-keeping.”^4,19^ In future research on end-of-life care, it is important to involve relatives from the first conversation with the patient about their participation until the end of the study.

### Strength and limitations

Some limitations should be considered. We did not validate the self-developed questions, so the outcomes should be interpreted with caution. Further, selection bias may have played a role: mainly relatives with strong positive or negative experiences may have participated. One interview was a double interview: the account of their experiences could be mutually influenced. Furthermore, the timing of the interviews in relation to the patients’ death varied which may have an impact on bereaved relatives’ memories and feelings about the patient’s dying process. Finally, we did not study the experiences of relatives whose loved one decided not to participate in the RCT.

### Implications

The SILENCE study was performed to assess the effect of prophylactic administration of scopolamine butylbromide on death rattle.^14^ The additional questionnaire and interview study indicated that the majority of the relatives of patients who participated in this RCT, did not experience burden or stress due to participation. Most of these relatives appreciated this research. RCTs at the end of life seem possible, if the patient and relatives are carefully and repeatedly informed about the study. It is important to not to lose sight of the important role of relatives in the decision-making concerning participation in research.

### Conclusion

Asking patients to participate in a double-blind placebo-controlled medication trial at the end of life is feasible. Patients and relatives jointly decided about participation in this research. The results of this study can be an incentive to initiate more RCTs to inform evidence-based care at the end of life. Informed consent procedures for these types of studies should allow patients to make well-considered decisions about participation together with their relatives.

## Supplemental Material

sj-pdf-1-pmj-10.1177_02692163221127557 – Supplemental material for When a dying patient is asked to participate in a double-blind, placebo-controlled clinical trial on symptom control: The decision-making process and experiences of relatives Click here for additional data file. Supplemental material, sj-pdf-1-pmj-10.1177_02692163221127557 for When a dying patient is asked to participate in a double-blind, placebo-controlled clinical trial on symptom control: The decision-making process and experiences of relatives by Harriëtte J van Esch, Arianne Stoppelenburg, Lia van Zuylen, Carin CD van der Rijt and Agnes van der Heide in Palliative Medicine

sj-pdf-2-pmj-10.1177_02692163221127557 – Supplemental material for When a dying patient is asked to participate in a double-blind, placebo-controlled clinical trial on symptom control: The decision-making process and experiences of relatives Click here for additional data file. Supplemental material, sj-pdf-2-pmj-10.1177_02692163221127557 for When a dying patient is asked to participate in a double-blind, placebo-controlled clinical trial on symptom control: The decision-making process and experiences of relatives by Harriëtte J van Esch, Arianne Stoppelenburg, Lia van Zuylen, Carin CD van der Rijt and Agnes van der Heide in Palliative Medicine

sj-pdf-3-pmj-10.1177_02692163221127557 – Supplemental material for When a dying patient is asked to participate in a double-blind, placebo-controlled clinical trial on symptom control: The decision-making process and experiences of relatives Click here for additional data file. Supplemental material, sj-pdf-3-pmj-10.1177_02692163221127557 for When a dying patient is asked to participate in a double-blind, placebo-controlled clinical trial on symptom control: The decision-making process and experiences of relatives by Harriëtte J van Esch, Arianne Stoppelenburg, Lia van Zuylen, Carin CD van der Rijt and Agnes van der Heide in Palliative Medicine
