# Supplementary material for: When a dying patient is asked to participate in a double-blind, placebo-controlled clinical trial on symptom control: The decision-making process and experiences of relatives
Source: Palliat Med. 2022 Dec 12;36(10):1552–8. doi: 10.1177/02692163221127557 (PMC9749009; doi:10.1177/02692163221127557)
Supplement: sj-pdf-1-pmj-10.1177_02692163221127557 – Supplemental material for When a dying patient is asked to participate in a double-blind, placebo-controlled clinical trial on symptom control: The decision-making process and experiences of relatives [file sj-pdf-1-pmj-10.1177_02692163221127557.pdf]

Appendix 1. Questionnaire

A. General questions

A1 What is your age? ……….years

A2 I am:
0 male
0 female

A3 In general, how is your health?
0 very good
0 good
0 it goes well
0 sometimes good, sometimes bad
0 bad

A4 What relationship did you have with the patient?
0 I was his/her partner
0 I was his/her child
0 I was his/her parent
0 other (you can fill in your relationship here) ……………………….

A5 What is the patient's date of birth?
… day … month …… year

A6 When did your loved one die?
… day … month …… year

A7 What was his/her nationality?
0 Dutch
0 Other (you can fill in his/her nationality here) ………………………

A8 What was his marital status?
0 Married or cohabiting
0 widowed
0 divorced
0 single
0 otherwise

A9 Did the patient had children?
0 yes
0 no you can go to question A11

A10
What age is this child or are these children?
(more answers are possible)
0-12 years: number of children in this age range: ....
13-18 years: number of children in this age group: ....
19 years or older: number of children at this age: ....

A11 What was the patient's living situation?
0 alone
0 with partner
0 with partner and child/children
0 without partner with child/children
0 with parent(s)
0 otherwise

A12
What was his highest completed education?
0 elementary school
0 lower vocational education
0 secondary vocational education
0 higher vocational education
0 scientific education, university
0 otherwise

A13 Was the patient religious?
0 yes
0 no, you can go to question B1
0 I don't know

A14 What faith / religion did the patient had?
0 Roman Catholic
0 Protestant
0 Islamic
0 Buddhist
0 Hindu
0 Other (you can fill in his religion or belief here) …………………..
0 I do not know

<u>B. Meaning and experience</u>

The following questions are about how you experienced your loved one's dying phase and the impact of participating in research "Death rattle in the dying phase: is prophylactic treatment useful?" (hereafter referred to as "the research") on it. There are also some questions that deal with participating in research in general.

B1.How burdensome was participation of your loved one in the study for yourself?
Not at all burdensome
A bit burdensome
Burdensome
Very burdensome

B2.How stressful was participation of your loved one in the study for yourself?
Not at all stressful
A bit stressful
Stressful
Very stressful

B3. How burdensome was participation of your loved one in the study for other relatives (children/siblings/friends)?

Not at all burdensome
A bit burdensome
Burdensome
Very burdensome

B4. How stressful was participation of your loved one in the study for other relatives (children/siblings/friends)?

Not at all stressful
A bit stressful
Stressful
Very stressful

B5. How burdensome do you think participation in the study was for your loved one?

Not at all burdensome
A bit burdensome
Burdensome
Very burdensome

B6. Has the quality of dying changed as a result of participation in the study?

Strongly improved
Improved
Unchanged
Deteriorated

B7. Do you find participation in research generally valuable?

Not at all valuable
A bit valuable
Valuable
Very valuable

B8. Would you participate in future research in the last phase of your life?

No
Yes
Do not know
