# Supplementary material for: When a dying patient is asked to participate in a double-blind, placebo-controlled clinical trial on symptom control: The decision-making process and experiences of relatives
Source: Palliat Med. 2022 Dec 12;36(10):1552–8. doi: 10.1177/02692163221127557 (PMC9749009; doi:10.1177/02692163221127557)
Supplement: sj-pdf-2-pmj-10.1177_02692163221127557 – Supplemental material for When a dying patient is asked to participate in a double-blind, placebo-controlled clinical trial on symptom control: The decision-making process and experiences of relatives [file sj-pdf-2-pmj-10.1177_02692163221127557.pdf]

Appendix 2. Interview guide

Introduction

Explain the purpose of the interview (the research focuses on the influence experienced by loved ones through the participation of their family member in research in the dying phase)

Check whether the respondent has read the information letter

Discuss confidentiality, audio recording

Opportunity to ask questions

Evaluation of the dying phase

How do you look back on the death of your loved one?

Prompts:

- Why do you consider this death as good/bad?
- What went well, what went less well, what could have been done better?

Were you informed about the dying phase?

Prompts:

- Was there a time when the doctor or other health care professional told you that your loved one was dying? How did you experience this?

Evaluation of research

Can you tell me about the course of the research?

Prompts:

- How was the decision to participate in the study made?
- Did you decide together or was the choice maid by your loved one?
- What was the motivation of your loved one to participate?
- How did you experience that decision? (difficult / easy / other)
- Were you informed about the start of the study medication?
- Did participation in this study influence dying or how you experienced it? Can you tell us how? (improved / deteriorated)
- What did it mean to you that your loved one wanted to participate in research?

Finally

How are you now?

Prompts:

- Grief counseling
- Physical and mental functioning
- Receive support after the patient's death
