# Supplementary figures and images for: When a dying patient is asked to participate in a double-blind, placebo-controlled clinical trial on symptom control: The decision-making process and experiences of relatives
Source: Palliat Med. 2022 Dec 12;36(10):1552–8. doi: 10.1177/02692163221127557 (PMC9749009; doi:10.1177/02692163221127557)

### Appendix 3. Code tree for analysis

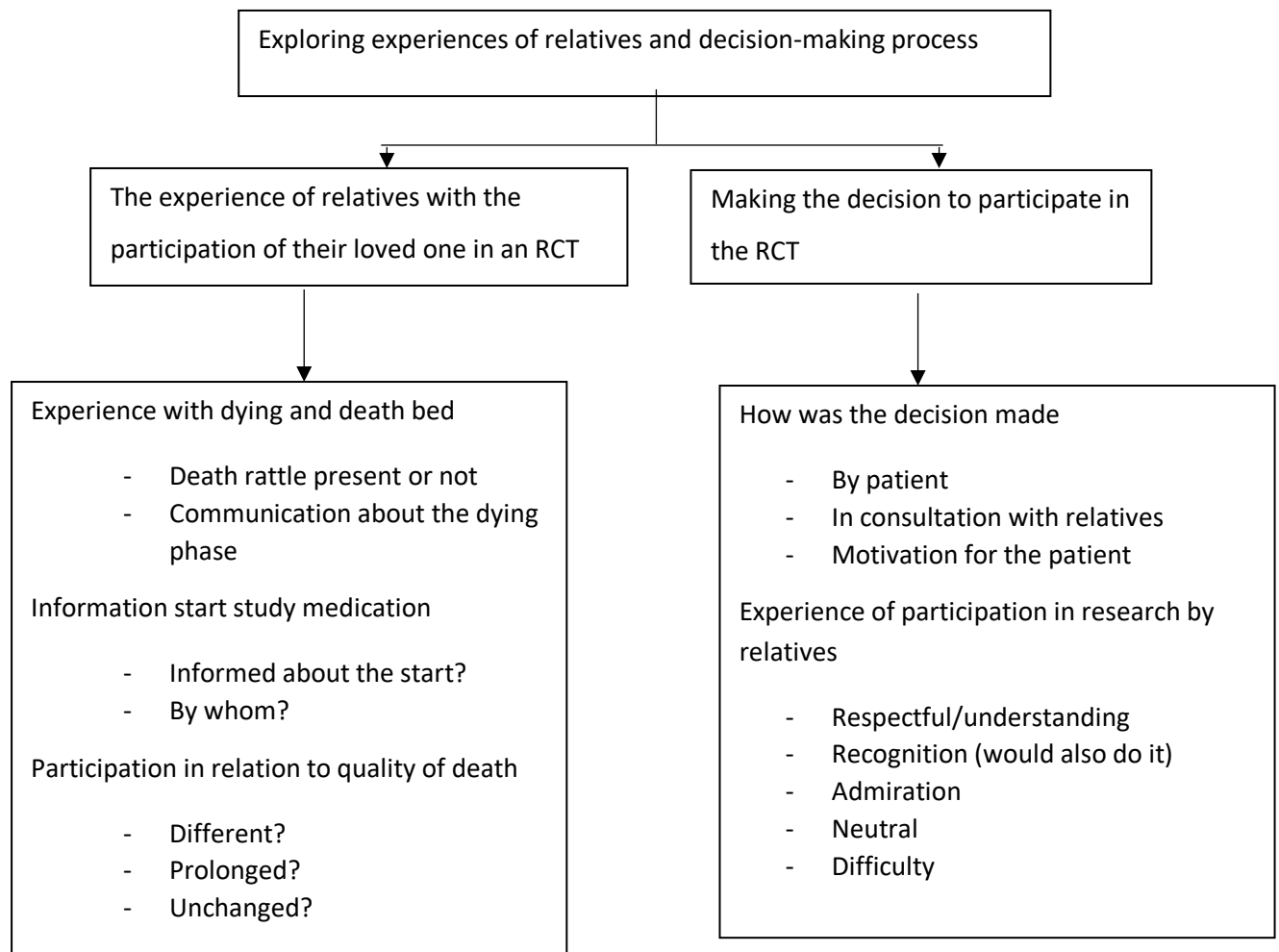

Supplement: sj-pdf-3-pmj-10.1177_02692163221127557 – Supplemental material for When a dying patient is asked to participate in a double-blind, placebo-controlled clinical trial on symptom control: The decision-making process and experiences of relatives [file sj-pdf-3-pmj-10.1177_02692163221127557.pdf]
